# Light-harvesting bio-nanomaterial using porous silicon and photosynthetic reaction center

**DOI:** 10.1186/1556-276X-7-400

**Published:** 2012-07-17

**Authors:** Kata Hajdu, Csilla Gergely, Marta Martin, László Zimányi, Vivechana Agarwal, Gabriela Palestino, Klára Hernádi, Zoltán Németh, László Nagy

**Affiliations:** 1 Department of Medical Physics and Informatics, University of Szeged, Szeged, H-6720, Hungary; 2 Laboratoire Charles Coulomb, UMR 5221 CNRS - Université Montpellier 2, Montpellier, F-34095, France; 3 Institute of Biophysics, Biological Research Centre of the Hungarian Academy of Sciences, Szeged, H-6701, Hungary; 4 CIICAP - Universidad Autonoma del Estado de Morelos, Col Chamilpa, Cuernavaca, 62209, Mexico; 5 Facultad de Ciencias Químicas, Universidad Autónoma de San Luis Potosí, San Luis Potosí, 78270, Mexico; 6 Department of Applied and Environmental Chemistry, University of Szeged, Szeged, H-6720, Hungary

**Keywords:** Porous silicon functionalization, Peptide, Photosynthetic reaction center, Nanomaterial, Biophotonics

## Abstract

Porous silicon microcavity (PSiMc) structures were used to immobilize the photosynthetic reaction center (RC) purified from the purple bacterium *Rhodobacter sphaeroides* R-26. Two different binding methods were compared by specular reflectance measurements. Structural characterization of PSiMc was performed by scanning electron microscopy and atomic force microscopy. The activity of the immobilized RC was checked by measuring the visible absorption spectra of the externally added electron donor, mammalian cytochrome *c*. PSi/RC complex was found to oxidize the cytochrome *c* after every saturating Xe flash, indicating the accessibility of specific surface binding sites on the immobilized RC, for the external electron donor. This new type of bio-nanomaterial is considered as an excellent model for new generation applications of silicon-based electronics and biological redox systems.

## Background

In the last few years, the use of bio-nanocomposites has been the subject of extensive study. Using a hybrid material, it may be possible to harness the advantages of two different materials at the same time. Several attempts to fabricate functional biocomposites by different groups have been reported [1-6]. Photosynthetic reaction center (RC) is one of the proteins of high interest, because it is nature's solar battery, converting light energy into chemical potential in the photosynthetic membrane, thereby assuring carbon reduction in cells [7,8]. Although RC functions on the nanometer scale, with nanoscopic power, this is the protein that assures the energy input practically for the whole biosphere on Earth. The extremely large quantum yield of the primary charge separation (close to 100%) [9] in RC presents a great challenge to use it in artificial light harvesting systems. However, as biological materials are very sensitive to the external effects and are generally stable only in their own environment, to keep them functional after their isolation, a special vehicle is necessary to hold and protect them from degradation.

Numerous investigations have recently focused on micro- and nanostructured materials due to the drastic increase in the surface area-to-volume ratio compared with the bulk materials. One of the promising nano-structured materials is porous silicon (PSi), well known for photonic applications, sensors, and novel drug delivery methods [10-15]. Various applications of PSi in bio-nanotechnology are possible due to its advantageous properties namely tunable pore dimensions, large surface area, multilayered photonic structures, easy and cheap fabrication method, and biocompatibility. The exceptional electrical and optical properties and the particular multilayered photonic structures offer unique application possibilities in integrated optoelectronic and biosensing (biophotonic) devices as well [10,13,14]. On the other hand, meso- and macroporous silicon assures good conditions for the penetration of the required biomolecules. The pore size and optical properties are adjustable during the wet electrochemical etching process, which is used to fabricate the well-arranged one-dimensional photonic structure [16].

In this work, RC was immobilized on the surface of porous silicon microcavities via two different methods: covalent binding and non-covalent attachment via a specific peptide interface (‘peptide binding’). In both cases, the RC preserved its activity, but the efficiency of the two methods turned out to be clearly different.

## Methods

### Sample preparation

*Rhodobacter sphaeroides* R-26 cells were grown photo-heterotrophically [17,18]. RCs were prepared by LDAO (*NN*-dimethyldodecylamine-*N*-oxide; Fluka AG, St. Gallen, Switzerland) solubilization and purified by ammonium sulfate precipitation, followed by DEAE Sephacel (Sigma-Aldrich Corporation, St. Louis, MO, USA) anion-exchange chromatography.

Porous silicon microcavity (PSiMc) structures were prepared by wet electrochemical etching process using boron-doped p^++^ type silicon wafers (thickness 500 to 550 μm) with a 0.002 to 0.004 ohm·cm resistivity, and with a crystallographic orientation of (100). Silicon substrates were etched at room temperature with an electrolyte consisting of HF (48%), ethanol (99.9%), and glycerol (99.99%), in the volumetric ratio of 3:7:1. Current densities of 85 and 40 mA/cm^2^ were used to produce high and low porosity layers, respectively. As-etched PSi samples were thermally oxidized at 800°C [16].

Binding of RC protein within the PSiMc scaffold was performed via two different methods: (1) covalent binding through a three-step conjugation method with 3-aminopropyl-triethoxysilane (APTES) and glutaraldehyde (GTA) as cross-linker molecule [14] and (2) peptide functionalization. In the first method, silanization of the surface with APTES ensures free amine groups on the surface. Subsequent treatment by GTA, an amine-targeted homobifunctional cross-linker molecule, and finally by RC ensures covalent linkage between APTES and the protein. The second method (i.e., peptide functionalization) is based on the binding of RC to PSiMc by strong physical attachment through a hydrophobic peptide layer (SPGLSLVSHMQT). This peptide, elaborated via phage display technology, reveals a high and specific binding affinity for the p^++^ Si material [13].

### Atomic force microscopy

Atomic force microscopy (AFM) images were recorded in air, with an Asylum MFP-3D head equipped with a Molecular Force Probe 3D controller (Asylum Research, Santa Barbara, CA, USA). Images were acquired in tapping mode using rectangular silicon cantilevers with a tip radius smaller than 10 nm; typically 7 nm (Olympus Micro Cantilever, OMCL-AC240TS, Olympus Corporation, Shinjiku, Tokyo, Japan). Images were taken at 1 Hz scan rate and digitized in 512 × 512 pixels.

### Scanning electron microscopy

Scanning electron microscopy (SEM) was performed by a Hitachi S-4700 Type II FE-SEM (Hitachi High-Tech, Minato-ku, Tokyo, Japan) equipped with a cold field emission gun operating in the range of 5 to 15 kV. The samples were mounted on a conductive carbon tape and sputter-coated by a thin Au/Pd layer in Ar atmosphere prior to the measurement. Elemental analysis of the samples was performed using energy-dispersive X-ray spectroscopy (EDX) with a RÖNTEC XFlash Detector 3001 (Rontec Holdings AG, Berlin, Germany) coupled with a silicon drift detector.

### Specular reflectometry

Reflectivity spectra were recorded with a Bruker 66 V Fourier transform infrared spectrometer, using a Bruker A 510, 11° specular reflection unit (BRUKER AXS GMBH, Karlsruhe, Germany). The PSi samples were illuminated with the tungsten source, and the reflected beam was detected with the silicon diode detector. The resulting spectra were captured in the range of 25,000 to 9,000 cm^−1^ (400 to 1,100 nm) after each modification step of PSiMc structures. All spectra were averaged over 100 scans with a spectral resolution of 2 cm^−1^[13].

### Optical spectroscopy

The PSiMc sample containing the bound RCs was placed in a 1 × 1-cm spectroscopic cuvette next to its rear wall facing the Xe flash beam. The cuvette was filled with buffer (10 mM TRIS, pH 8.0, 100 mM NaCl, 0.03% LDAO) containing reduced horse heart cytochrome *c* (Sigma-Aldrich) as electron donor and UQ-0 (2,3-methoxy-5-methyl-1,4-ubiquinone; Sigma-Aldrich) as electron acceptor for the light-activated RC. The oxidation of cytochrome *c* by RCs bound to PSi was checked by steady state absorption measurements using a UNICAM 4 spectrophotometer (Unicam Limited, Cambridge, UK). Cytochrome *c* was reduced by ascorbate before the experiments were conducted.

## Results and discussion

### Morphological characterization

**Figure 1:**
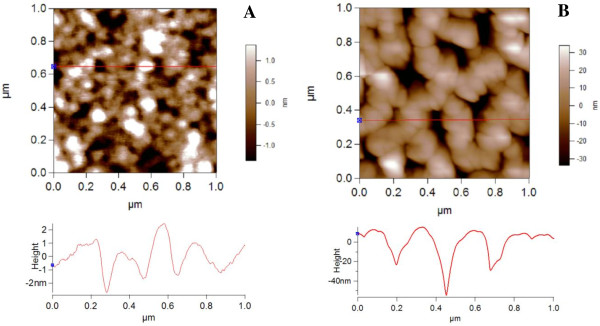
**AFM surface topography image (1 × 1 μm) and profile section.** The untreated PSiMc (**A**) and after RC binding (**B**).

Before binding RC to PSiMc, the surface structure of this supporting material was explored by AFM and SEM. The AFM image (Figure 1A) was obtained before any treatment of PSiMc surface, revealing a high porosity top layer, with a roughness (calculated as root mean square average (RMS)) of 882 ± 150 pm. The pore diameter sizes range from 50 to 90 nm.

**Figure 2:**
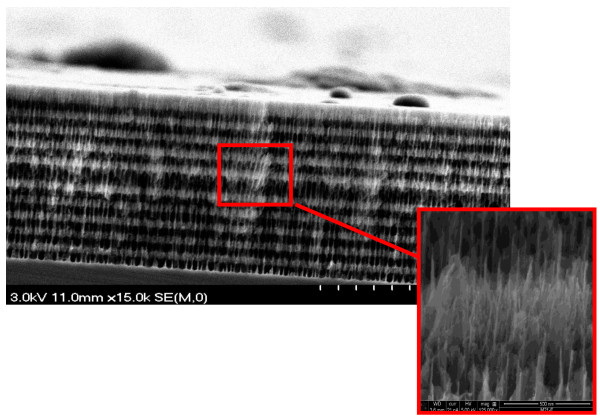
**SEM image revealing cross-sectional view of untreated multilayered structure (PSiMc) supported on bulk silicon substrate.** Dark and light regions correspond to the high and low porosity layers, respectively. Magnified view of the nanostructured multilayer is also shown.

The structure of the active surface of the PSiMc (before and after binding) was also investigated by SEM, extended by EDX analysis. Well-defined nanostructured Bragg type PSi multilayers with optical thickness = λ/4 on either side of the thick high porosity active layer in the middle (optical thickness = λ/2) are clearly visible in the SEM image (Figure 2).

### Binding efficiency measurements

**Figure 3:**
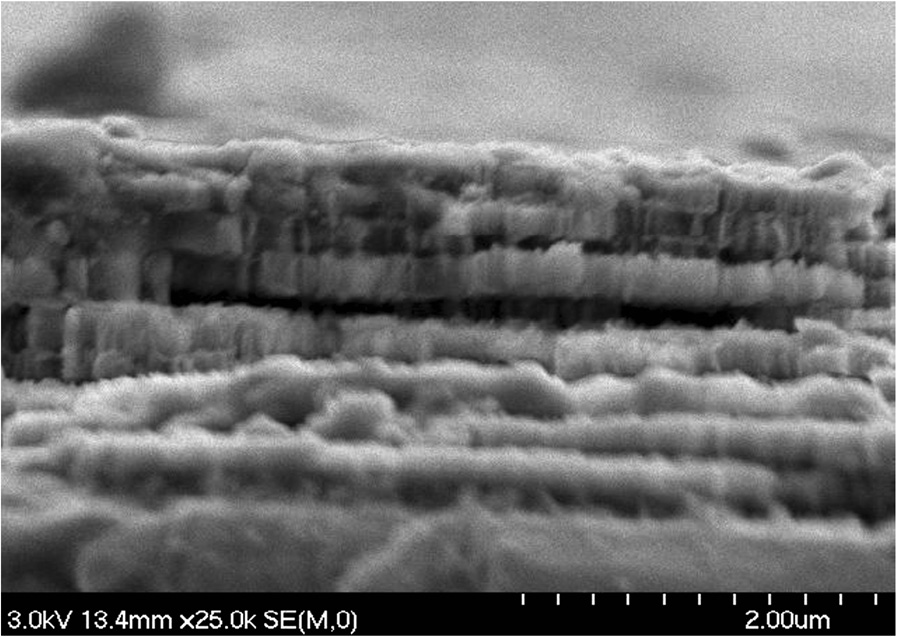
SEM image of PSiMc cross section after RC binding.

After the chemical procedure of the RC binding (surface oxidation, silanization, attachment of the RC protein through the GTA linker), the AFM image showed a more uniform slick surface (Figure 1B), with a higher surface roughness, RMS = 15.7 ± 0.5 nm. Moreover, due to the high porosity of the initial structure, the pores appeared to merge and form larger pores, with a diameter size ranging from 70 to 150 nm to allow the RC with approximately 10 nm in diameter [19] to penetrate more easily. The dramatic change in the surface appearance before and after RC protein binding, as the surface roughness increases, indicates good RC protein attachment to the PSi scaffold. SEM images also show that the layers are covered by large amounts of RC (Figure 3). It must be mentioned, and Figures 2 and 3 show, that the pore size is large enough (7 to 15 times the RC diameter) to overload the layers of the PSi by the protein under optimal conditions.

**Figure 4:**
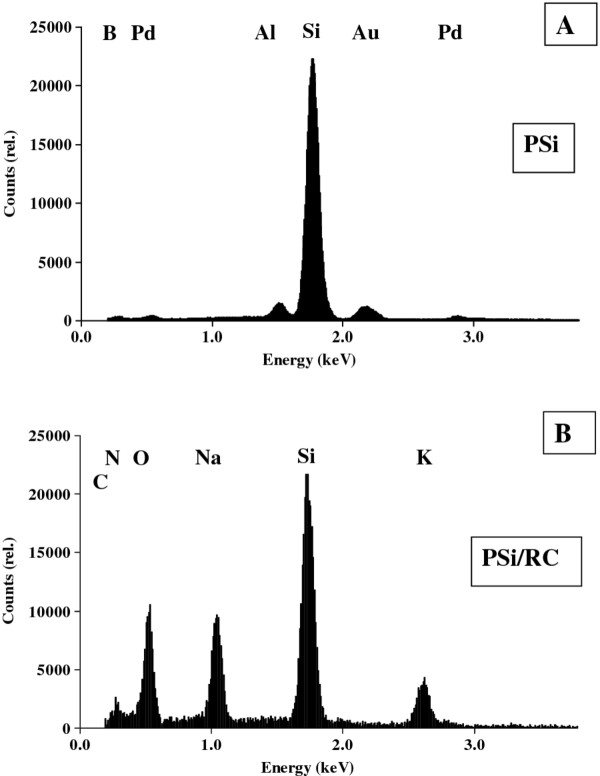
**Result of element analysis by EDX for (A) untreated PSi and (B) treated with 6.0-μM RC.** The corresponding elements found in the samples are also indicated.

Elemental composition analysis by energy dispersive X-ray spectroscopy (Figure 4) also proved RC binding to PSiMc. In the untreated PSiMc devices, the EDX spectrum showed the abundance of Si at any depth in the layer structure and the absence of the characteristic elements of the organic compounds, i.e., C, O, or N (Figure 4A). In RC treated samples, the contribution of C, N, and O were significant (Figure 4B), which confirms the protein infiltration.

Other elements such as Au, Pd, and Al were also detected to some extent. The gold, palladium, and aluminum signals originate from the sample pre-preparation phase (sputtering and sample holders).

**Figure 5:**
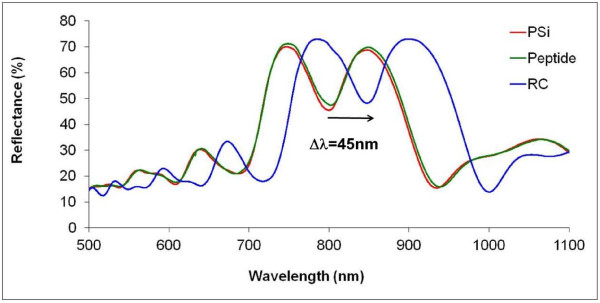
**Reflectance spectra of the PSiMc recorded at different steps of the treatment of the sample.** Arrow indicates the red shift induced in the photonic structure by the RC binding.

The reflectance spectra of the PSiMc were recorded after each modification step by the infrared spectrometer. Figure 5 compares spectra taken before and after the peptide functionalization and finally after the RC binding to the surface of PSiMc. Protein binding causes a red shift in the reflectance spectra that depends on the amount of the bound molecules.

**Figure 6:**
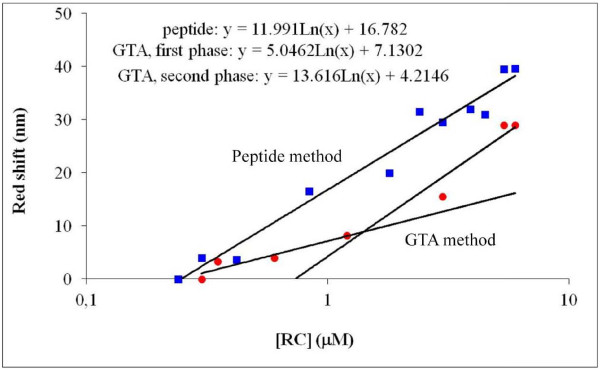
**Magnitude of red shifts of resonance peaks as a function of incubation concentration of RC.** Squares show the data of the peptide; circles, those of the GTA method.

Figure 6 shows the efficiency of RC binding via the two different methods. Line A (peptide method) runs above line B (GTA method) at any RC concentration, indicating that at the same concentration of RC, the red shift was always larger in the case of the peptide method than with the GTA method. It appears that the RC has bigger affinity to the peptide-coated surface than to the silanized samples, across the GTA cross-linker, so it is easier to form a physical binding than a chemical one. On the other hand, as the peptide coating generates a more homogenous and hydrophobic surface, the RC can also coat the surface quite homogeneously. Earlier investigations have shown that the high hydrophobicity of membrane proteins seems to be an important factor for better adsorption and functional characteristics in nanopores of FSM (folded-sheet mesoporous silica material) [20,21], which is probably the case in PSi as well. A successful application of specific oligopeptides for supporting protein binding to PSi was described recently [13].

The binding of RC to PSiMc can be modeled by saturation characteristics so that it follows straight lines in a logarithmic representation. The saturation curve for the GTA method might indicate a slight biphasic character as compared with the strictly monophasic behavior with the peptide method. The slope of the fitted line is about two times larger (12.0 nm) for the peptide method compared with the one found for the initial phase (5.0 nm) and almost the same as the second phase (13.6 nm) of the GTA method. Hence, it can be concluded that the binding affinity of the RCs to the peptide-coated PSi is about twice as large as to the GTA.

### RC photochemistry

The overall electron transfer through the RC in living organisms and in reconstituted systems is coupled to the oxidation of cytochrome *c*_2_ (the native electron donor) on the donor side and to the redox cycle of quinones on the acceptor side of the protein. Direct optical detection of cytochrome photooxidation in the cytochrome cycle is a reliable method of tracking the steps of the RC photocycle [22,23]. The oxidation of cytochrome *c* can be followed by the change in the spectra, i.e., the gradual decrease in the absorption mainly at 550 nm after every flash excitation.

**Figure 7:**
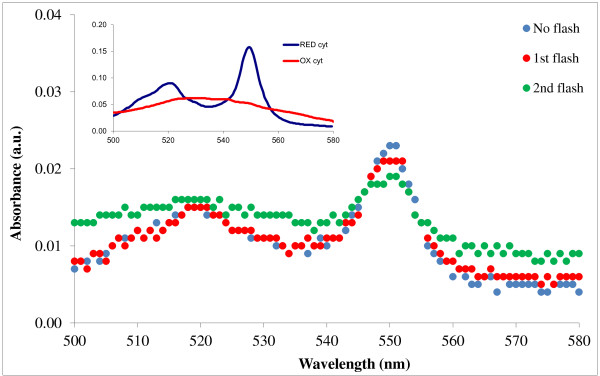
**Change in cytochrome*c*spectra in the aqueous medium induced by RC/PSiMc complex after light excitation.** Inset: absorption spectra of oxidized and reduced cytochrome *c*.

Figure 7 shows the change in the cytochrome spectra after every flash excitation, induced by the immobilized RC. Inset of Figure 7 shows the spectra of the fully oxidized and fully reduced cytochrome for comparison. In control experiments, no spectral change could be observed when the RC/PSiMc complex was removed from the cuvette, indicating that illumination alone could not induce cytochrome *c* oxidation in the solution.

Cytochrome *c* oxidation shows that the RC photocycle could be restored after reconstitution of the donor and acceptor sites with cytochrome *c* and UQ, respectively. Hence, the specific binding sites of the PSi-bound protein stayed accessible for these externally added agents.

## Conclusions

Successful infiltration of reaction center into PSiMc photonic structure and the retention of its photochemical activity were demonstrated. After reconstitution of the donor and acceptor sites, the RC photocycle was also restored, i.e., the accessibility of the secondary quinone site and of the cytochrome binding site was not blocked in the PSiMc matrix. This functional integrity is promising in terms of further research into the properties and applicability of this photo excitable semiconductor biophotonic material containing the photosynthetic reaction center, an exceptionally efficient natural light harvesting system.

## Competing interests

The authors declare that they have no competing interests.

## Authors’ contributions

KH carried out the main experimental work. CG designed the work. MM made AFM images of the samples. LZ completed physical characterization of the complexes. VA and GP prepared and characterized the PSiMc samples. KH made chemical characterizations of the complexes. ZN took SEM and EDX images of the samples. LN organized the manuscript. All authors read and approved the final manuscript.
